# DNA Metabarcoding of Amazonian Ichthyoplankton Swarms

**DOI:** 10.1371/journal.pone.0170009

**Published:** 2017-01-17

**Authors:** M. E. Maggia, Y. Vigouroux, J. F. Renno, F. Duponchelle, E. Desmarais, J. Nunez, C. García-Dávila, F. M. Carvajal-Vallejos, E. Paradis, J. F. Martin, C. Mariac

**Affiliations:** 1 Institut de Recherche pour le Développement, Université de Montpellier, Unité Mixte de Recherche Diversité Adaptation et Développement des Plantes (UMR DIADE), Montpellier, France; 2 Institut de Recherche pour le Développement, Unité Mixte de Recherche Biologie des Organismes et Ecosystèmes Aquatiques (UMR BOREA), MNHN—CNRS-7208—UPMC—UCBN—IRD-207, Montpellier, France; 3 Laboratoire Mixte International—Evolution et Domestication de l’Ichtyofaune Amazonienne (LMI—EDIA), IIAP—UAGRM—IRD, UMR BOREA, 7 rue Cuvier, CP 32, Paris, France; 4 Institut des Sciences de l’Évolution (UMR ISEM), Université Montpellier—CNRS—IRD—EPHE, Place Eugène Bataillon—CC 065, Montpellier, France; 5 Instituto de Investigaciones de la Amazonía Peruana (IIAP), Laboratorio de Biología y Genética Molecular (LBGM), Iquitos, Perú; 6 FAUNAGUA, ULRA-UMSS, ECOSINTEGRALES SRL, Cochabamba, Plurinational State of Bolivia; 7 Montpellier-SupAgro, Centre de Biologie pour la Gestion des Populations (UMR CBGP), Montferrier-sur-Lez, France; Xiamen University, CHINA

## Abstract

Tropical rainforests harbor extraordinary biodiversity. The Amazon basin is thought to hold 30% of all river fish species in the world. Information about the ecology, reproduction, and recruitment of most species is still lacking, thus hampering fisheries management and successful conservation strategies. One of the key understudied issues in the study of population dynamics is recruitment. Fish larval ecology in tropical biomes is still in its infancy owing to identification difficulties. Molecular techniques are very promising tools for the identification of larvae at the species level. However, one of their limits is obtaining individual sequences with large samples of larvae. To facilitate this task, we developed a new method based on the massive parallel sequencing capability of next generation sequencing (NGS) coupled with hybridization capture. We focused on the mitochondrial marker cytochrome oxidase I (*COI*). The results obtained using the new method were compared with individual larval sequencing. We validated the ability of the method to identify Amazonian catfish larvae at the species level and to estimate the relative abundance of species in batches of larvae. Finally, we applied the method and provided evidence for strong temporal variation in reproductive activity of catfish species in the Ucayalí River in the Peruvian Amazon. This new time and cost effective method enables the acquisition of large datasets, paving the way for a finer understanding of reproductive dynamics and recruitment patterns of tropical fish species, with major implications for fisheries management and conservation.

## Introduction

The Amazon basin is thought to hold 30% of all river fish species described to date [1]. This exceptional wealth of fish species synchronize their reproduction to benefit from the highly predictable annual flooding regime of the Amazon basin (flood pulse concept, [2]). Annual flooding increases food availability for fish larvae and juveniles, and facilitates their dispersal [3,4]. Inundated floodplains provide also shelter for both parents and juveniles [2, 4–5]. For migratory fish, the exceptional dimension and complexity of the Amazonian hydrographic network provide a large number of dispersion routes for eggs and larvae [6,7]. Consequently, the study of their recruitment is difficult. These migratory fish species represent over 80% of fishery catches in the Amazonian basin, and most of them belong to the orders Characiformes and Siluriformes (catfish) [8–10]. Some of the most important catfish species, in particular the largest species of the genus *Brachyplatystoma*, are already overexploited [11–15]. Among this genus, *Brachyplatystoma rousseauxii* undertakes the longest migration known in freshwaters, 5500 kilometres [16]. The adults reproduce in the piedmont of Andean rivers in Bolivia, Colombia, Ecuador and Peru. The larvae then drift downstream to their nursery area in the Amazon estuary where they grow before migrating upriver to join the breeding adults in the headwaters [14–18]. This long-distance migratory life cycle is threatened by human activities, particularly overfishing and the construction of hydroelectric dams, which could drastically limit larval recruitment, hence the renewal of fisheries resources. Understanding the reproductive cycles and recruitment dynamics of commercially important migratory species is thus essential for the sustainable management of fisheries in Amazonia. Regular, standardized plankton net sampling of the larvae of these species is a promising way to achieve these goals. One of the technical limits of this approach is the specific identification of freshwater fish larvae, particularly those of Siluriformes, given the great number of species in this order. Morphological criteria alone in early development stages only enable differentiation of families, and sometimes of genera, but very rarely of species [19,20]. With the development of dedicated databases and the standardization of molecular tools, molecular approaches like DNA barcoding offer a solution to this problem. The technique has already been successfully used to identify adult siluriform species [21–23]. More recently this approach was extended to the identification of larvae [24,25] using individual Sanger sequencing. This approach is very reliable, but it can quickly become burdensome when several thousand larvae need to be identified. We therefore developed an alternative strategy using high throughput sequencing of batches of larvae. Our main objectives were to evaluate whether this approach is able to 1) retrieve all the different species in a mixture of larvae, and 2) quantify the relative proportions of species in the batch. PCR based methods can lead to biased amplification of DNA from divergent species and artificially lower the representation in the final counts [26–28]. Consequently, we developed a hybridization-based method to capture a commonly used DNA barcode marker: a portion of the cytochrome oxidase 1 (*COI*) gene. The mitochondrial molecular marker *COI* (600 bp) was selected for its ability to distinguish fish [29–32]. Our approach also enables the simultaneous analysis of several batches of larvae, hence further reducing sequencing effort. Moreover, it is very easy to extrapolate from a proof-of-concept on a single marker to several markers, whole mitochondria, or even several thousand nuclear genes [33].

## Materials and Methods

### Study area and sampling

In 2013 and 2014, larvae (ichthyoplankton) were sampled in three tributaries of the Amazon River: the Napo, Ucayalí and Marañon in Peru (for a list of samples, see S1 Table). A partner of the French National Institute of Research for Sustainable Development (IRD), the *Instituto de Investigaciones de la Amazonia Peruana* (IIAP) is rightfully authorized by the Peruvian government to study and to sample the Peruvian Amazonian biodiversity. The DIREPRO office "*Dirección Regional de la Producción del Gobierno Regional de Loreto*" authorized the export of fish larvae to IRD laboratories in France. Ichthyoplankton were sampled in daylight by towing an ichthyoplankton net behind a boat with an outboard motor maintained in a static position. The protocol used 1.5 m long conical-cylindrical nets with an aperture diameter of 0.6 m, and a mesh size of 1 mm or 0.3 mm depending on the sample, each net containing a collector cup in its end. Either one net was used, or three nets arranged vertically, with a distance of 2 m between each. The nets were towed for 15 min, five times a day, over a period of two days. Larvae were fixed in a 96% ethanol solution and dried just before DNA extraction following the procedure published by Sambrook *et al*. [34].

### Larval sample

We used two samples, one from the Napo River and one from the Marañon River, to validate the method. The two samples of larvae were randomly divided into two subsets. One subset was used for DNA extraction from each individual larva (270 larvae from the Marañon and 102 larvae from the Napo). Part of the extracted DNA was pooled in equimolar quantities to form two samples named Mar-mock-NGS (for the Marañon larvae) and Nap-mock-NGS (for the Napo larvae). In the second subset, total DNA was extracted using all larvae together for the Marañon (250 larvae) and for the Napo (373 larvae) to form two bulk samples, Mar-bulk-NGS and Nap-bulk-NGS for the Marañon and Napo larvae, respectively.

Each larva from the first subset was individually Sanger sequenced. We named the results of the Sanger sequencing of Napo and Marañon Nap-S and Mar-S, respectively. The mock and the bulk samples were sequenced using an Illumina platform.

### *COI* amplification and sanger sequencing

A 680 bp fragment of the *COI* gene was amplified using universal primers Fish F1 and Fish R1 and PCR conditions as previously described by Hubert *et al*. [29]. PCR was performed with a Phusion® High-Fidelity PCR Master Mix (ThermoFisher, 1040–2678) using the following program: 3 minutes at 98°C followed by 30 cycles of 80 seconds at 98°C, 45 seconds at 55°C and one minute at 68°C, with a final extension of 10 minutes at 72°C. PCR products were sequenced using the BigDye Terminator 3.1 kit (Applied Biosystems). Sequencing was performed on an ABI prism 3130 (Applied Biosystems), at IRD Montpellier, (France) using the Fish F1 primer. Sequences are available on Dryad doi:10.5061/dryad.117tn (see S2 Table).

### Library preparation

Preparation of biotinylated PCR probes for capture. *COI* probes were produced by PCR using the same conditions as above but with 5’ biotinylated primers synthesized by Eurogentec SA (Angers France). Amplification was performed on four adults from four different species: *Oxydoras niger* (oni), *Pimelodella sp*. (pgra), *Pinirampus pirinampu* (ppi) and *Pseudoplatystoma punctifer* (ppu). The four species were selected to cover the panel of siluriform species present in our database, spanning a nucleotide divergence range of 20%. PCR probes were purified and pooled at equimolar concentrations for capture.

Library preparation and capture were performed as described in Mariac *et al*. [33]. Libraries were tagged using 6-bp oligonucleotides (TAG) to allow for multiplexing, and then hybridized with *COI*-targeted probes as described in Mariac *et al*. [33]. Briefly, total DNA was sheared using a Covaris E220 sonicator (Covaris, Woburn, Massachusetts, USA), end-repaired, ligated and nicks were filled in before pre-hybridization PCR was performed. These products were mixed and hybridized with biotin-labeled probes. The streptavidin-biotinylated probes-target complexes were magnetically immobilized during a 3 min. denaturation step at 96°C to elute the enriched library fraction. A non-enriched library was also produced as control using genomic DNA from a subset of the Napo 2013 sample. This library was used to assess the efficiency of the capture method. The concentrations of the libraries were quantified by qPCR (Kappa Biosystems) after which paired-end sequencing was performed using MiSeq v2 reagents and 2 × 150 bp on an Illumina MiSeq v3 platform at the CIRAD facilities (Montpellier, France).

### Data analysis

We built a database with sequences of *COI* genes from 86 adult siluriform species, referenced in different collections or publications (see S5 Table) and for which the phylogenetic relationships are established and discussed in Carvajal et al. [35]. The 86 sequences of *COI* used were deposited in GenBank (see S5 Table). This database contains only species of economic importance in Amazonia and consequently does not include all the genera of Siluriformes. The database allowed species identification of the samples by mapping sequences to those in the reference database.

The sequences produced using the Sanger method were examined with Geneious Pro 4.8.5 software [36]. Automatic filtering involved trimming regions with more than a 5% chance of error per base. Sequences were manually checked and any ambiguities corrected. The sequences were aligned with the reference database using MUSCLE (100 iterations, [37]). Finally, species assignment was computed by building a neighbor-joining tree using Tamura-Nei genetic distances.

The sequences obtained with the NGS method were demultiplexed using the script “demultadapt” (available at https://github.com/Maillol/demultadapt), that sorts reads according to their TAG (6 bp, 0 mismatch threshold). Adapters were removed using Cutadapt 1.2.1 software with the following parameters: length overlap = 7 bp, insert size = 35 bp. Reads were trimmed based on their nucleotide mean quality (Q < 30) using the script “filter-mean-quality” (available at https://github.com/SouthGreenPlatform/arcad-hts/blob/master/scripts/arcad_hts_2_Filter_Fastq_On_Mean_Quality.pl). Sequences were then mapped on the reference database using the BWA mem v.0.7.12 package [38,39]. For each enriched library, we calculated 1) the percentage of useful reads, i.e. the ratio of the number of reads mapped on the database to the total number of reads, and 2) X-fold enrichment, which corresponds to the ratio of the number of reads from the enriched library mapped to the reference library to the number of reads from the non-enriched (genomic) library mapped to the reference library. X-fold measures the effectiveness (the enrichment) of the experiment. The percentage of useful reads measures the specificity of the enrichment.

As we had built up the bulk DNA from species we were able to identify using Sanger sequencing, we knew which species we expected to find using the NGS approach. We were thus able to identify false positives, i.e. species found using the NGS approach that were not present in the bulk DNA. Only properly paired reads with a maximum of one mismatch, no clipping, and no secondary alignment with the reference were used. Different filters were applied on the alignments: only the sequences for which both the R1 and R2 reads were mapped to the same species on the database were kept, and we kept only those mapped with quality > 0 (MAPQ>0), only sequences with strictly less than two mismatches, and an alignment over 100 bp. A given species was identified by listing which reference sequences the filtered reads were mapped to with a minimum coverage of 60%, otherwise we considered that it was not sufficiently precise for species level identification and kept only the genus taxonomic rank. The frequency of each identified species was calculated using the relative number of mapped reads divided by the total number of mapped reads across species. Finally, the composition and frequencies obtained from Sanger sequencing and from NGS sequencing were compared to validate the capture method. Pearson's product-moment correlations were calculated with R 3.2.5 [40].

### Diversity study

Here, we describe a case study in which the new sequencing method was assessed over a period of eight months. To estimate the composition of siluriform species in samples collected monthly in the Ucayalí River between March and October 2014 (see S1 Table), we performed bulk DNA extraction, and used the NGS capture method on the eight samples to obtain the sequence data.

## Results

### Identification of species composition

**Fig 1:**
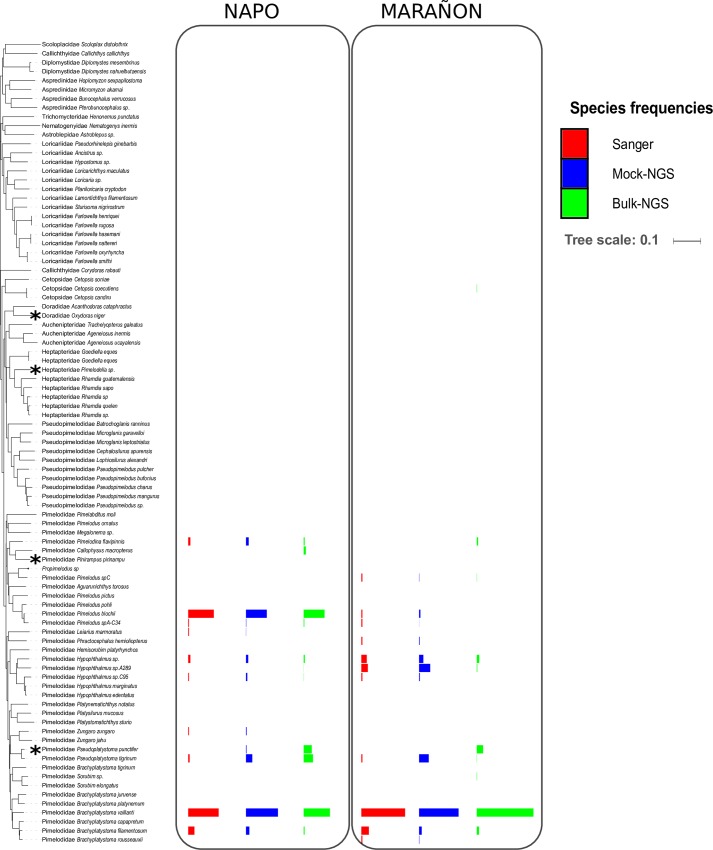
Neighbor joining tree built with 89 sequences of Pimelodidae species. The 89 sequences correspond to all the individuals present in our database, and the tree was built according to the Tamura-Nei model. Stars identify the four species used for the production of PCR probes, and the color bars show the proportions of each species in the two samples produced using the two sequencing methods.

The *COI* marker was successfully Sanger sequenced for 270 and 102 larvae from the Marañon and Napo rivers, respectively. A total of 168 sequences corresponded to siluriform species. A total of 158 sequences were very close or identical to the 86 species of our database. Ten sequences presented some divergence from these 86 species. These ten sequences were redundant, and were narrowed down to three different sequences. These three sequences were added to our dataset, as we thought they might either represent three described species not yet sequenced or three new species. They were named *Pimelodidae_Pimelodus_spA-C34*, *Pimelodidae_Hypophthalmus_sp*.*C95* and *Pimelodidae_Hypophthalmus_sp*.*A289*. The final database thus contained 89 sequences (Fig 1). Based on the revised reference dataset, the Sanger method identified 11 different species in the Marañon River sample (Mar-S) and 10 different species in the Napo River sample (Nap-S) (Table 1).

**Table 1:** Data on the alignment of the *COI* sequences for the Napo and Maranon samples. The table summarizes the results and estimated frequencies of each species identified using next generation sequencing (NGS) and the Sanger approach. For the next generation approaches, a "Mock" was prepared with equimolar DNA from each larva and a "Bulk" with DNA extracted from a batch of larvae. The number of reads, the percentage of mismatches, the percentage of base coverage, the average coverage depth, maximum coverage and estimated frequency are given for each species. For the Sanger approach (Nap-S, Mar-S), the number of individuals sequenced and the frequency of the species is given. Species are denoted including the family name “Family_Genus_species”. When coverage was considered low (below 60%), the data are underlined and the identification is only given at the genus level (denoted Family_Genus_(species)_spX).

**Species**	**Mar-Mock-NGS**						**Mar-Sanger**		**Mar- Bulk-NGS**					
	Number of reads	Mismatch (%)	Bases with coverage (%)	Average coverage depth	Maximum coverage depth	Estimated frequency (%)	Individuals	Frequency (%)	Number of reads	Mismatch (%)	Bases with coverage (%)	Average coverage depth	Maximum coverage depth	Estimated frequency (%)
Pimelodidae *Brachyplatystoma vaillanti*	1,128	0.2	99.3	234.4	718.0	56.1	41	62.1	1,933	0.3	99.3	401.8	1,204.0	80.5
Pimelodidae *Hypophthalmus sp*.	123	0.2	97.5	24.8	85.0	6.1	5	7.6	84	0.2	97.7	17.3	54.0	3.5
Pimelodidae *Pseudoplatystoma tigrinum*	277	0.1	98.7	58.7	108.0	13.8	1	1.5	5	0.1	37.2	1.0	5.0	0.2
Pimelodidae *Brachyplatystoma filamentosum*	76	0.1	100.0	16.0	37.0	3.8	7	10.6	76	0.1	96.5	16.0	35.0	3.2
Pimelodidae *Brachyplatystoma rousseauxii*	8	0.1	67.0	1.7	5.0	0.4	1	1.5	0					0.0
Pimelodidae *Pimelodus blochii*	38	0.4	73.9	8.0	27.0	1.9	1	1.5	0					0.0
Pimelodidae *Phractocephalus (hemioliopterus) spX*	17	0.0	42.6	3.4	16.0	0.8	1	1.5	0					0.0
Pimelodidae *Hypophthalmus sp*.*C95*	21	0.2	85.0	4.7	10.0	1.0	1	1.5	0					0.0
Pimelodidae *Pimelodus (spC) spX*	6	0.1	28.9	1.3	6.0	0.3	1	1.5	7	0.1	45.3	1.4	6.0	0.3
Pimelodidae *Pimelodus (spA-C34) spX*	1	0.7	24.1	0.2	1.0	0.1	1	1.5	0					0.0
Pimelodidae *Hypophthalmus sp*.*A289*	317	0.2	100.0	71.1	114.0	15.8	6	9.1	18	0.2	98.0	3.9	9.0	0.7
Pimelodidae *Pseudoplatystoma punctifer(70)*	0					0.0	0	0.0	215	0.2	98.7	45.2	89.0	9.0
Pimelodidae *Pimelodina flavipinnis(46)*	0					0.0	0	0.0	44	0.3	81.6	9.1	22.0	1.8
Pimelodidae *Sorubim sp*.*(76)*	0					0.0	0	0.0	10	0.5	65.5	2.1	6.0	0.4
Cetopsidae *Cetopsis coecutiens(126)*	0					0.0	0	0.0	10	0.3	68.2	2.0	5.0	0.4
TOTAL	2,012					100.0	66	100.0	2,402					100.0
**Species**	**Nap-Mock-NGS**						**Nap-Sanger**		**Nap-Bulk-NGS**					
	Number of reads	Mismatch (%)	Bases with coverage (%)	Average coverage depth	Maximum coverage depth	Estimated frequency (%)	Individuals	Frequency (%)	Number of reads	Mismatch (%)	Bases with coverage (%)	Average coverage depth	Maximum coverage depth	Estimated frequency (%)
Pimelodidae *Brachyplatystoma vaillanti*	1,551	0.2	99.3	322.8	851.0	45.5	44	43.1	2,132	0.2	99.3	441.7	1,483.0	37.3
Pimelodidae *Pimelodus blochii*	1,009	0.5	98.8	211.1	635.0	29.6	37	36.3	1,691	0.5	98.8	351.0	1,137.0	29.6
Pimelodidae *Brachyplatystoma filamentosum*	164	0.1	100.0	34.5	83.0	4.8	9	8.8	76	0.1	87.3	16.0	36.0	1.3
Pimelodidae *Hypophthalmus sp*.	107	0.1	98.8	21.9	64.0	3.1	3	2.9	91	0.1	94.0	18.3	80.0	1.6
Pimelodidae *Pimelodina flavipinnis*	131	0.3	93.4	26.9	76.0	3.8	3	2.9	80	0.3	100.0	16.4	58.0	1.4
Pimelodidae *Pseudoplatystoma tigrinum*	305	0.1	98.8	63.9	103.0	8.9	2	2.0	752	0.1	98.8	158.7	237.0	13.2
Pimelodidae *Zungaro zungaro*	34	0.0	74.3	7.2	22.0	1.0	1	1.0	0					0.0
Pimelodidae *Leiarius (marmoratus) spX*	7	0.1	41.3	1.5	7.0	0.2	1	1.0	0					0.0
Pimelodidae *Hypophthalmus sp*.*C95*	63	0.1	94.6	13.3	26.0	1.9	1	1.0	8	0.2	35.9	1.7	8.0	0.1
Pimelodidae *Pimelodus spA-C34*	13	0.2	74.8	3.0	6.0	0.4	1	1.0	53	0.1	90.1	12.4	22.0	0.9
Pimelodidae *Pseudoplatystoma punctifer*	27	0.3	76.8	5.8	13.0	0.8	0	0.0	656	0.2	98.8	137.9	230.0	11.5
Pimelodidae *Callophysus macropterus(21)*	0					0.0	0	0.0	173	0.6	99.3	35.6	101.0	3.0
Pimelodidae *Hypophthalmus sp*.*A289(184)*	0					0.0	0	0.0	1	0.7	22.5	0.2	1.0	0.0
TOTAL	3,411					100.0	102	100.0	5,713					100.0

### Enrichment in *COI* sequences

X-fold enrichment ranged from 71 to 247-fold (see S1 Table), meaning that we found 71 to 247-fold more sequences from the target *COI* region after enrichment. After capture, the percentages of reads that mapped to the target region ranged from 0.29% to 1.03%.

### Comparison of species identification by the NGS vs. the sanger method

The enrichment approach using the mock samples provided results that were largely congruent with those obtained with the Sanger sequencing method. The species compositions of the Marañon and Napo samples inferred from the mock-NGS capture method were identical to those obtained using the Sanger sequencing approach, which is considered as the reference (Table 1). Even species with very low frequency, such as *Zungaro zungaro* (0.98%) in the Nap-S sample and *Brachyplatystoma rousseauxii* (1.54%) in the Mar-S sample, were identified with the NGS capture method. We also identified a false positive in the NAP-mock-NGS sample (Table 1). This species, *Pseudoplatystoma punctifer*, only represented 0.79% (27 reads) of the total number of reads.

The initial bulk of larvae was divided into two bulks and, as expected, low frequency species varied between the two bulks. Seven species not present in the Sanger reference were identified in the Marañon (4) and Napo (3) bulk-NGS samples. Conversely, five species in the Marañon sample and two in the Napo sample were present in the Sanger sample but were not retrieved in the respective bulk-NGS samples. The frequency of all these species was < 1.5%.

We would like to emphasize here that we successfully identified distant species with our four probes. The species *Hypophthalmus sp*.*C95*, which was identified in the Marañon and Napo mock samples using the NGS capture method, was the most genetically distant species [41] in the four probes (see S3 Table). This species presented 87.6% pairwise identity with the closest probe designed from *Pseudoplatystoma punctifer*, implying that the probes are able to capture very distant targets, even if the species is present at a low frequency (0.98%) in the sample.

### Analysis of estimated frequencies

**Fig 2:**
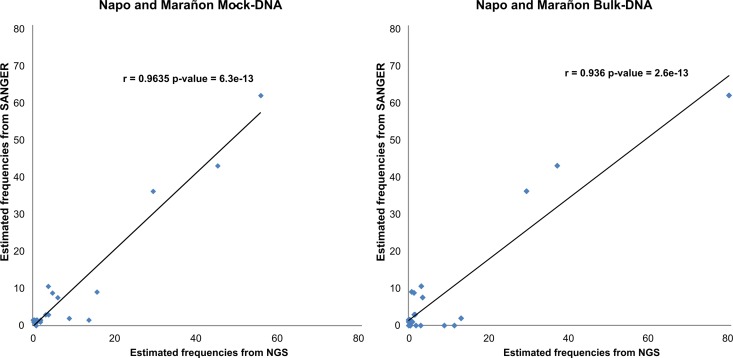
Validation of estimated species frequencies. Correlation between frequencies of species as a function of the sequencing method. The frequencies estimated with the Sanger method and the frequencies estimated with the Mock-NGS and Bulk-NGS methods from the Napo and Maranon samples were correlated.

Species frequencies were very well correlated between Sanger sequencing and the bulk of larvae. Strong significant correlations were found between Sanger and NGS frequencies: r = 0.96 (p < 0.001) for the mock-NGS and r = 0.94 (p < 0.001) for bulk-NGS (Fig 2).The samples were characterized by one or two dominant species: in Mar-S, *B*. *vaillantii* represented 62% of the species and in Nap-S, *B*. *vaillantii* plus *Pimelodus blochii* represented 79.4% of the species present (Table 1). The frequencies of the remaining species rapidly dropped below 10%.

### Application to temporal variability of the samples

**Fig 3:**
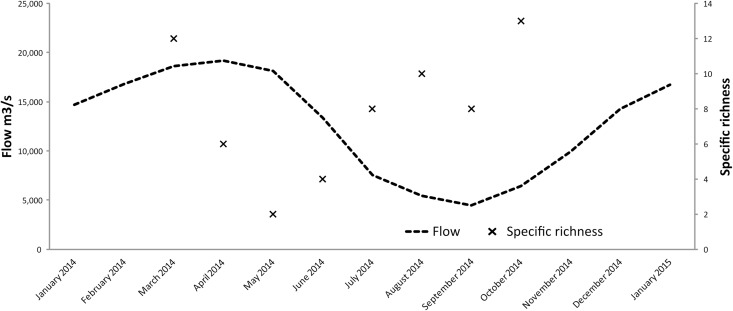
Changes in water flow and in specific richness over time. Flow and specific richness were compared in the Ucayalí River in March and October 2014. Flow values came from records at the Requena site (id 10074800) on the Ucayalí River. Flow data were provided by the SO-HYBAM (Observation Service—Geodynamical, hydrological and biogeochemical control of erosion/alteration and material transport in the Amazon basin) and SENAMHI (*Servicio Nacional de Meteoloría e Hidrología)*, Peru.

Changes in species richness over the eight month study period (Fig 3) tended to correlate with the hydrological cycle: species diversity was lower during high water and falling water periods, and higher during low water and rising water periods. Each batch was represented by one or two main species, plus a number of species present at lower frequencies. The number of species identified in each sample ranged from 2 to 13.

Detailed examination of species composition (see S4 Table) showed that the species belonged to two groups: those present almost all year round, such as *B*. *vaillantii* and *B*. *filamentosum*; and those present for only a few months, such as *P*. *pirinampu*.

## Discussion

The aim of this study was to validate and use a new approach to study the specific diversity of siluriform larvae in river plankton samples. We validated the method and provided evidence for its reliability. Overall, the NGS approach made it possible to identify and quantify the species composition in a swarm of larvae in each sample. The enrichment rate was high, i.e. a 71 to 247-fold increase, and in agreement with observations in the literature [42–44]. The percentage of useful reads is still relatively low, but this likely reflects the size of our target sequence relative to the whole genome. Although the rate of useful reads is low, it is nevertheless cost effective and labor saving. Considering only the sequencing cost (library preparation # US$10 per sample can be disregarded), sequencing 300 larvae in each of 30 samples (9000 larvae) at US$5 per larva (Sanger sequencing) is very expensive compared to a single run of MISEQ sequencing at US$1,100 for the same 30 samples. Several strategies have been proposed to increase the percentage of useful reads including two rounds of capture [45]. This would be a useful improvement but it needs to be tested to see if the double capture does not bias the estimation of species frequencies.

Comparing the Sanger method and the NGS method enabled us to validate the qualitative estimation of species composition, although we also found one false positive *P*. *punctifer* in the bulk Nap-NGSeq sample, which we knew from the Sanger method was not present. This species is genetically very closely related to *P*. *tigrinum*, which was also found in the sample (97.8% pairwise identity on 685 nucleotides). Consequently, the false positive could be the consequence of mapping to a very similar sequence. Longer reads might improve mapping precision in the future.

The two samples from the Marañon and Napo rivers were randomly but equally divided into two halves, one for the Sanger (Mar-S and Nap-S) and equimolar (mock-NGS) analyses and the other for the non-equimolar analyses (bulk-NGS). When we compared the Sanger or mock-NGS with the bulk-NGS, we observed differences in the presence of low frequency species (<1.5% in the Sanger sample). This is not surprising, as species found once or at a low frequency in a whole sample may be found in only one of the split samples. Overall, predictability of the relative frequency of species was very high. For low frequency species (below 10%) quantification might include some over- and underestimation. This might result from the variability in standardization or the difference in genome size between species, both of which lead to variation in the targeted *COI* sequences between larvae.

One limit worth mentioning is the need for a reference database to map our NGS sequences. Here we focused on commercially important and hence widely distributed species. Our reference contained 86 species of Siluriformes, which does not represent the whole species diversity of Siluriformes, but we only added three putative species to our database out of 564 Sanger sequenced larvae. In a few cases, we were only able to identify a larva to the genus level. As new species are sequenced, the database will be enriched and the precision of identifications improved.

Using our database, we were able to assess the temporal and spatial dynamics of species reproduction in the Ucayalí River. The results showed that the breeding activity of the Pimelodidae family was spread out over the eight months of the study. However, the results also suggested that more species reproduce during the falling and rising water periods, which is consistent with the results of earlier studies on adults [14,17] and on larvae [25,46]. Together with the work of García-Dávila *et al*. [25], who used individual Sanger sequencing, this study allows the first assessment of the life cycle of several siluriform species analyzed together. Until now, the life cycle of only one species, *B*. *rousseauxii* [14] had been studied in the Peruvian Amazon. Although more samples will improve our understanding of this dynamics, this first study provides very interesting insights into both diversity dynamics and the reproductive activity of some species at specific periods. Our NGS metabarcoding method, which enables the analysis of several thousand larvae in a bulk sample, opens new opportunities for studying the breeding cycles and recruitment of fish species in Amazonia. Indeed, with specially designed sampling strategies, it will be possible to estimate the breeding seasons, the spawning grounds and the recruitment patterns of a large number of commercially important migratory siluriform species. As creating probes is simple and can be used on phylogenetically distant species, as shown by Liu *et al*. [47], this method could also be extended to other groups, such as Characiformes and Gymnotiformes. It could also be extended from a single gene to a whole mitochondrial genome.

## Conclusion

Compared to the classical Sanger method, here we demonstrate the reliability of our new metabarcoding method with enrichment by capture using probes. Our approach led to both specific identification of larvae in swarms and to the quantification of their relative abundance. The NGS approach has the potential to greatly improve our understanding of fish recruitment, and thus help develop adequate fisheries management and conservation strategies for Amazonian fish species. In addition, considering the alarming trend in the planning and construction of hydroelectric dams in the Amazon basin [48–50], quantifying the relative contribution of major sub-basins to the recruitment of important fish species might help guide decisions made by stakeholders and preserve river connectivity.

## Supporting Information

S1 Supporting Information NMDS ordination of inferred larval frequencies, March-October 2014, 8 months. (PDF)

S1 Table Details of samples used. (XLSX)

S2 Table List of Sanger sequences used in this study. (XLSX)

S3 Table Matrix of genetic distance between probes and identified species. (XLSX)

S4 Table Monthly frequency of species sampled in the Ucayalí River from March to October. (XLSX)

S5 Table Sequences (89) used in the reference database with corresponding GenBank ID. (XLSX)

S1 Text Command lines and softwares used. (SH)
