# Supplementary material for: DNA Metabarcoding of Amazonian Ichthyoplankton Swarms
Source: PLoS One. 2017 Jan 17;12(1):e0170009. doi: 10.1371/journal.pone.0170009 (PMC5241143; doi:10.1371/journal.pone.0170009)
Supplement: S1 Supporting Information — (PDF) [file pone.0170009.s001.pdf]

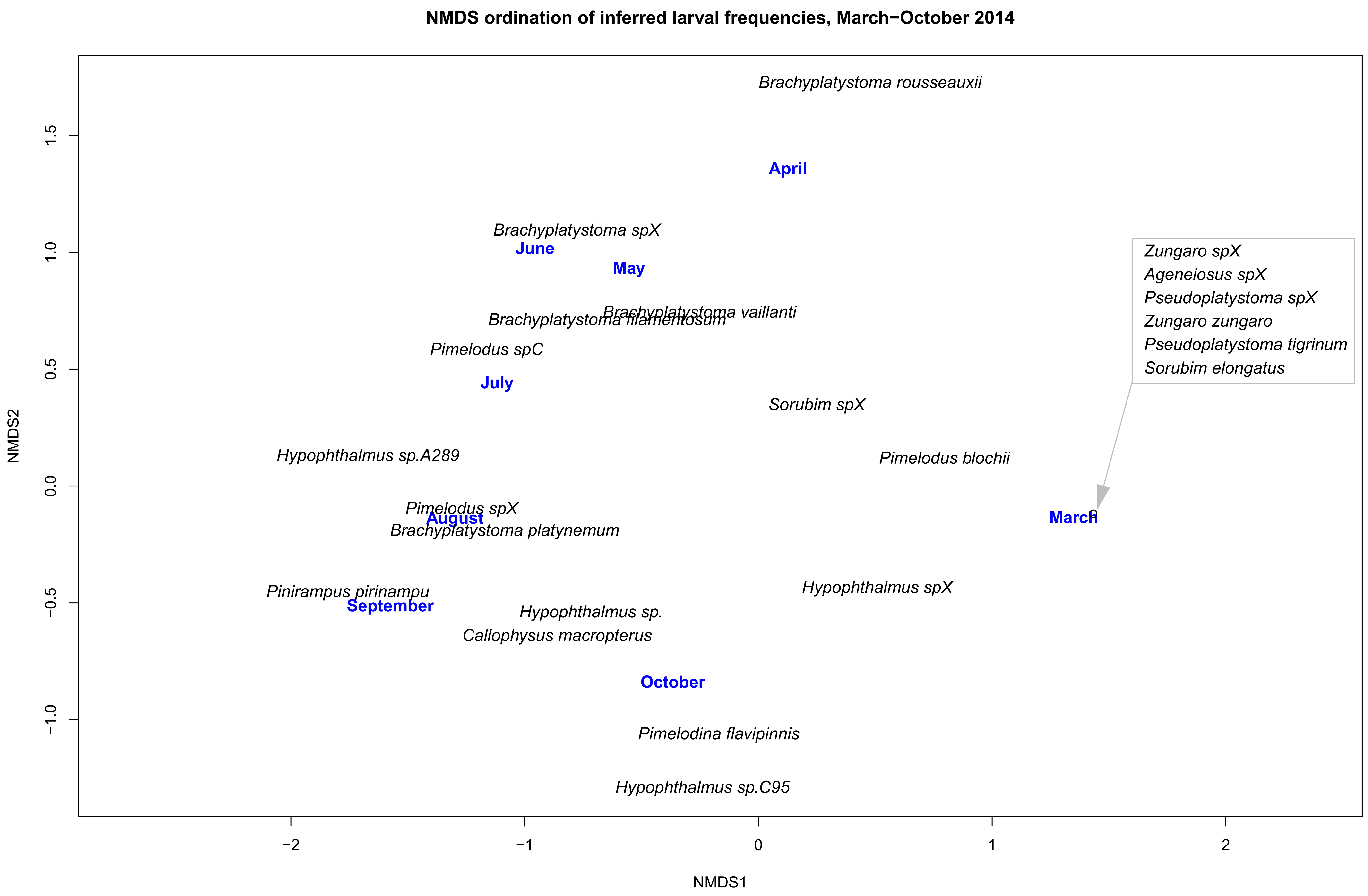

NMDS ordination of the larval sample across the 8 months. However, the figure mainly illustrates the most abundant species in each month and specific composition differences between high and low waters, information already provided in Fig 3 and S4 Table. The six species in the gray square have identical profiles and have the same coordinate (shown by the grey arrow).
